# The Relationship Between the Gut Microbiome and Neurodegenerative Diseases

**DOI:** 10.1007/s12264-021-00730-8

**Published:** 2021-07-03

**Authors:** Xueling Zhu, Bo Li, Pengcheng Lou, Tingting Dai, Yang Chen, Aoxiang Zhuge, Yin Yuan, Lanjuan Li

**Affiliations:** 1 State Key Laboratory for Diagnosis and Treatment of Infectious Diseases, National Clinical Research Center for Infectious Diseases, Collaborative Innovation Center for Diagnosis and Treatment of Infectious Diseases, The First Affiliated Hospital, Zhejiang University School of Medicine, Hangzhou, 310003 China; 2 Research Units of Infectious Disease and Microecology, Chinese Academy of Medical Sciences, Beijing, 100730 China; 3 The First Affiliated Hospital, Zhejiang University School of Medicine, Hangzhou, 310003 China

**Keywords:** Gut microbiome, Neurodegenerative diseases, Aging, 16S rRNA sequencing, Multi-omics, Microbiome-based therapies

## Abstract

Many recent studies have shown that the gut microbiome plays important roles in human physiology and pathology. Also, microbiome-based therapies have been used to improve health status and treat diseases. In addition, aging and neurodegenerative diseases, including Alzheimer's disease and Parkinson's disease, have become topics of intense interest in biomedical research. Several researchers have explored the links between these topics to study the potential pathogenic or therapeutic effects of intestinal microbiota in disease. But the exact relationship between neurodegenerative diseases and gut microbiota remains unclear. As technology advances, new techniques for studying the microbiome will be developed and refined, and the relationship between diseases and gut microbiota will be revealed. This article summarizes the known interactions between the gut microbiome and neurodegenerative diseases, highlighting assay techniques for the gut microbiome, and we also discuss the potential therapeutic role of microbiome-based therapies in diseases.

**Supplementary Information:**

The online version contains supplementary material available at 10.1007/s12264-021-00730-8.

## Introduction

In recent years, there has been increasing research interest in gut microorganisms and numerous papers have been published. A large number of studies have linked intestinal microbiome disorders to a variety of diseases, including inflammatory bowel disease (Crohn's disease [1], irritable bowel syndrome [2], and colon cancer [3]), neurological diseases (Alzheimer's disease (AD) [4], and Parkinson's disease (PD) [5]), metabolic diseases (diabetes [6] and obesity [7]), and musculoskeletal diseases (rheumatoid arthritis [8], osteoporosis [9], and gout [10]). Currently, several research efforts are exploring the links between intestinal microbiota and diverse diseases with the hope of new diagnostic and therapeutic approaches in addressing these diseases [11].

Because the purpose of modern medicine is to extend human life, the aging and neurodegenerative diseases that occur naturally during the aging process have long been of interest, and are currently receiving increasing research and clinical attention. Previous studies have shown that aging is not an irreversible process and that some of the life-extension mechanisms in some simple organisms have proved to be feasible anti-aging treatments for humans; however, these treatments cannot treat cognitive impairment [12]. Although numerous research efforts on neurodegenerative diseases are underway, the pathogenesis and treatment of many of them are still unresolved [13].

**Fig. 1:**
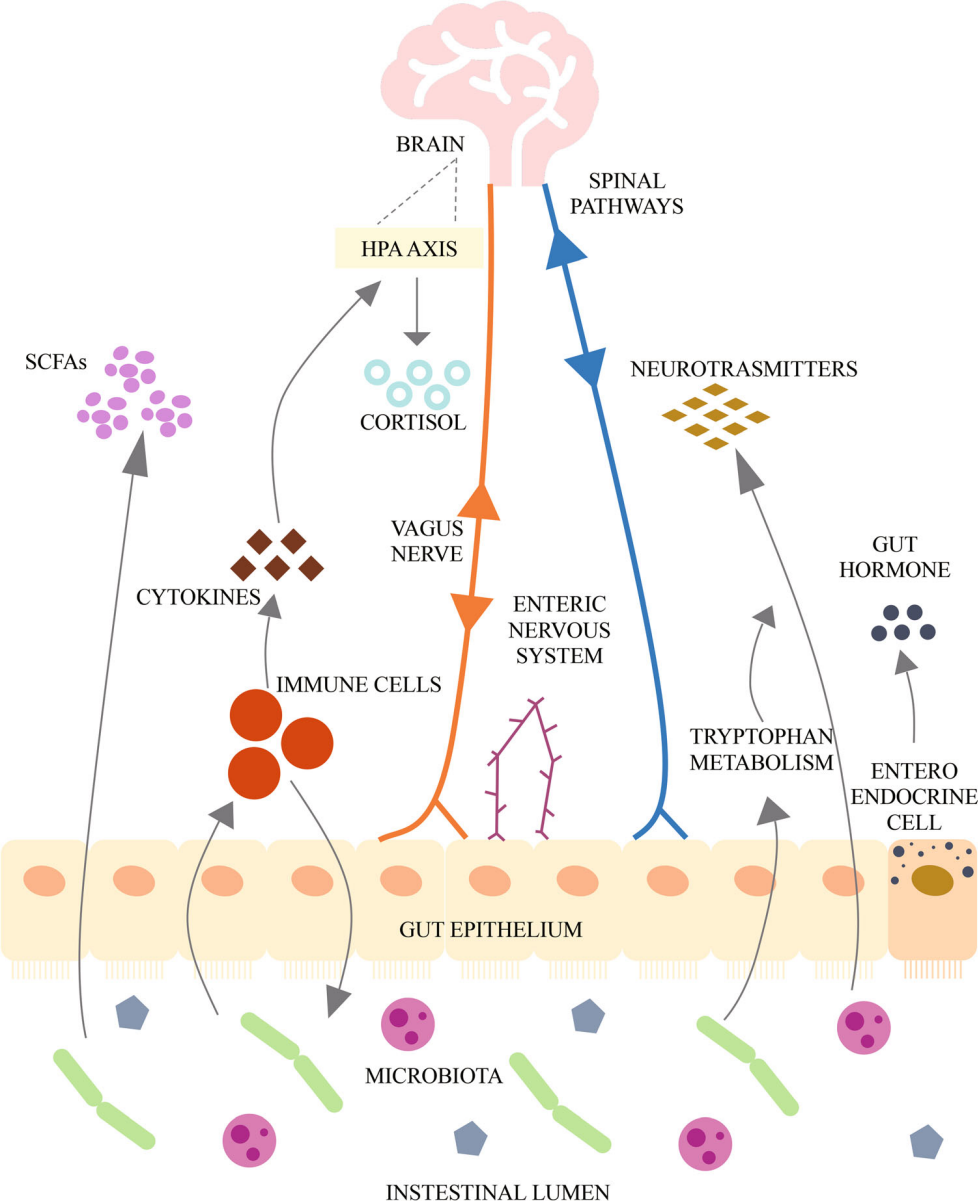
Key pathways in the brain-gut-microbiota axis. Key metabolites of gut microbes include short chain fatty acids (SCFAs), such as acetate and butyrate, which can play important roles in regulating the brain and behavior through G protein coupled receptors. Cytokines produced by intestinal immune cells can regulate the brain by activating the hypothalamic-pituitary adrenal (HPA) axis and releasing cortisol. The vagus nerve mediates bidirectional communication between the gut microbes and the brain. Signals from the brain are transmitted back to the enteric nervous system via the spinal cord or vagus nerve to control the intestine. Intestinal microbes can also secrete neurotransmitters (such as norepinephrine, gamma-aminobutyric acid, serotonin, and dopamine, etc.) that can communicate with the brain. Intestinal endocrine cells can secrete intestinal hormones and act on the brain. [14, 98]

The brain-gut-microbiome axis is a two-way communication system that allows gut microbes to communicate with the brain and the brain with the intestine. Changes on either side of this axis may cause changes to the other. The signaling between these two systems and the microbiome are complex, involving metabolic, immune, neuronal, and endocrine signaling pathways (Fig. 1), and thus far have not been fully elucidated [14]. It is known that the older the person, the more likely he or she is to suffer complications related to the gut or gut bacteria [15]. A growing number of researchers are working to decipher the specific connections between intestinal microbes and natural diseases of aging, and the expect to reveal the “secret recipe” for the prevention and treatment of diseases, and even longevity.

In this article, we aimed to investigate the relationship between the gut microbiome and neurodegenerative diseases, and to summarize the existing information on various methods for the detection and treatment of intestinal microbes, and to summarize the role of gut microorganisms in the diagnosis, treatment, and prevention of neurodegenerative diseases.

## The Relationship between the Gut Microbiome and Neurodegenerative Diseases

### Alzheimer's Disease (AD)

AD, generally known as dementia or cognitive impairment, is a typical degenerative disease of the central nervous system in the elderly, accounting for 60%-80% of all dementias [4]. Its feature is a progressive decline in cognitive function [16]. The neuropathology of AD is characterized by the deposition of amyloid β (Aβ), followed by the formation of hyperphosphorylated tau protein, which compose plaques and neurofibrillary tangles [17]. These deposits can trigger neuroinflammation, giving rise to synapse loss and neuronal death [18]. A clinical trial conducted on patients with AD found that amyloid-positive patients demonstrated a lower abundance of *Eubacterium rectale* and *Bacillus subtilis* and a higher abundance of *Escherichia*/*Shigella* in their stools compared to other groups, indicating the role of both amyloid and relevant bacterial accumulation in cognitive impairment [19]. It has been hypothesized that some components of the intestinal microbiota, such as *B. subtilis* and *E. coli*, secrete large amounts of lipopolysaccharides and amyloid proteins, [20], which may directly traverse the intestinal barrier or blood-brain barrier damaged by aging or disease, and/or exert an indirect effect to pass through these protective physiological barriers by lipopolysaccharide/amyloid-induced cytokines or other small pro-inflammatory molecules, leading to the development of AD [21]. The microbiome of the elderly with AD shows a lower proportion of bacteria synthesizing butyrate that contributes to anti-inflammatory activity and immunity regulation, as well as greater abundance of taxa that are known to cause pro-inflammatory states. Therefore, a potential therapy of AD is to modulate intestinal homeostasis by decreasing inflammatory, and increasing anti-inflammatory, microbial metabolism [22]. New research has found that fecal microbiota transfer therapy can ameliorate amyloidosis, tau pathology, reactive gliosis, and cognitive impairment in AD mice, which might be associated with a reversal of abnormalities in circulating blood inflammatory monocytes and in the colonic expression of genes associated with macrophage activity [23].

### Parkinson's Disease (PD)

PD is recognized as the second most common neurodegenerative disease, a movement disorder that is estimated to affect 1-2 out of every 1,000 people worldwide [14, 24]. PD patients also often suffer from non-motor symptoms, the most common of which is gastrointestinal dysfunction [25]. PD is characterized histopathologically by a remarkable depletion of dopaminergic neurons in the substantia nigra pars compacta, resulting in dopamine deficiency in the striatum, while intracellular eosinophilic inclusions (so-called Lewy neurites and Lewy bodies) are visible in the remaining neurons [24]. Alpha-synuclein (α-syn) aggregates, the main neuropathological markers of PD, are present in the submucosal and myenteric plexus of the enteric nervous system before being detected in the brain, which may indicate a spread of the disease from gut to brain [26]. The pathogenesis of PD may also relate to intestinal inflammation. Metabolites of the intestinal microbiota may trigger an immune response that induces intestinal inflammation and even the development of PD [27]. Sequencing of intestinal microbiota has revealed that the relative abundance of *Enterobacteriaceae* in the feces of PD patients is strongly correlated with the severity of postural instability and gait difficulties compared to controls [28]. In addition, lower levels of the intestinal hormone ghrelin, which is involved in regulating the activity of nigrostriatal dopamine, are associated with increased abundance of *Lactobacillaceae* and decreased abundance of *Prevotellaceae* in the gut microbiome [24]. The Gram-negative *Prevotellaceae* are involved in increasing mucin synthesis in the intestinal mucosal layer. Therefore, a decreased abundance of *Prevotellaceae* may lead to decreased mucin synthesis and increased intestinal permeability, resulting in more exposure to bacterial antigens and endotoxins, which may trigger excessive α-syn expression in the colon and even in the brain [29]. Also, the microbiome of PD patients is characterized by a decreased abundance of butyrate-producing bacteria with an increased abundance of pro-inflammatory *Proteobacteria*, which may trigger inflammation‐induced misfolding of α‐syn [30]. Osteocalcin ameliorates the motor deficits and dopaminergic neuronal loss in PD mice through increasing the potential of microbial propionate production and activating free fatty-acid receptor 3 in enteric neurons [31].

### Amyotrophic Lateral Sclerosis (ALS)

ALS is a progressive neurodegenerative disease that is associated with the death of brain and spinal motor neurons [32]. The prominent features of ALS are microglial activation and chronic neuroinflammation [33]. The symptoms of ALS include muscle weakness, muscle stiffness, muscle spasms, muscle twitching, cramps, and coordination problems, which lead to speech, swallowing, and breathing difficulties [32]. A clinical study of ALS patients found that gastrointestinal symptoms precede neurological symptoms, and examination of feces demonstrated that the diversity of intestinal microbiota is lower in ALS patients than in healthy controls [33]. Another clinical study reported changes in the composition of gut microbiome in ALS patients, including a significant decrease in the *Firmicutes*/*Bacteroidetes* ratio along with a decrease in the relative abundance of *Anaerostipes*, *Oscillibacter*, and *Lachnospiraceae*. This suggests that a pro-inflammatory gut microbiome disorder may disrupt the intestinal epithelial barrier, promote an immune/inflammatory response, and alter bowel motility [34]. Some researchers have hypothesized that intestinal barrier dysfunction facilitates the entry of toxins from the intestinal lumen to the blood, causing an increase in circulating lipopolysaccharides and an innate immune response, which plays a vital role in the pathogenesis of ALS [35]. A metabolite of the gut microbiome, nicotinamide, improves the motor symptoms and gene expression patterns in ALS mice, and nicotinamide is reduced systemically and in the cerebrospinal fluid in ALS patients [36].

### Huntington's Disease (HD)

HD is a progressive brain disease caused by amplification of the trinucleotide cytosine-adenine-guanine repeat sequence in the Huntington gene [37]. This mutation produces polyglutamine-expanded huntingtin protein, causing neuropsychiatric symptoms, cognitive impairment, and involuntary choreiform movements [38]. HD is one of the most fatal inherited neurodegenerative diseases without effective drug treatment [38]. Clinical studies have reported that a distinct serum metabolic profile, thought to originate from gut microbe-derived metabolites, is present in pre-symptomatic HD subjects and early symptomatic HD subjects, compared to controls, pointing to a potential role for the microbiome in the progression of HD [39]. Multi-omics integration analysis of HD mice suggests that the gut microbiome modulates the pathogenesis of HD by altering plasma metabolites [40]. These findings may provide clinically useful biomarkers for the onset, progression, and phenotypic variability of HD.

### Aging

Human aging is an inherent physiological process in which organs, including brain, gut, and intestinal microbiota, gradually decay over time [24]. Aging can also be considered as a low-grade chronic pro-inflammatory state, so-called ‘inflammaging’ [41], demonstrating a link between immune cells and aging [42]. Although there is little to no neurodevelopment after the onset of adulthood, aging still has an essential impact on central nervous system and intestinal functions. Aging may detrimentally affect the composition of the intestinal microbiota, which in turn may adversely affect human health [43]. It is known that the diversity and stability of intestinal microbes progressively decreases with age. Although members of the *Firmicutes* and *Bacteroidetes* continue to dominate the aging intestine, the relative proportions of these bacterial taxa may change. In the intestine, the numbers of beneficial bacteria decrease while populations of pathogenic bacteria increase. For example, *Bifidobacterium* and butyrate-producing bacteria (e.g., *Ruminococcus* and *Faecalibacterium*) decrease in numbers, while bacteria that stimulate inflammatory responses (e.g., *Enterobacteriaceae* and *Clostridioides difficile*) increase [44]. Age-related changes in gut microbial communities can induce physiological changes that are capable of altering immune system homeostasis and the inflammatory state, thereby increasing the risk of disease [45]. The health-related microbiota *Bifidobacterium*, *Akkermansia*, and *Christensenellaceae* have been found in the gut of extremely old people (105-109 years old) [46].

In conclusion, clear relations between the gut microbiome and neurodegenerative diseases have been identified. Gut microbes may accelerate the development of neurodegenerative diseases by eliciting autoimmunity and producing metabolites. Correspondingly, the composition of gut microbes can be modulated to alleviate diseases.

## Techniques for Assessment of the Gut Microbiome

### 16S rRNA Sequencing

16S rRNA sequencing is a well-established, reliable, and relatively inexpensive method for measuring the relative abundance of microorganisms using next-generation sequencing techniques [47] of samples from the gut microbiome of insects, animals, and humans [48]. It has been widely used to study the relationship between intestinal microbiota and various neurodegenerative diseases (Tables 1, 3, and Table S1). This technique uses the polymerase chain reaction to amplify genetic sequences present in the microbiota [47]. These amplified sequences (amplicons), have been clustered into operational classification units (OTUs) according to their genomic relationships so that their relative abundance in samples can be calculated. The methods for defining OTUs include *de novo* methods (where reads are useless outside the reference database) and closed-reference methods (where reads outside the reference database cannot be captured) [49].

**Table 1:** Representative research using 16S rRNA sequencing to test stool samples

Reference	Subjects	Condition	Main findings
Chi *et al.* [100]	Noise-exposed wild-type (WT) and APP/PS1 Tg Alzheimer's disease (AD) mice	AD	Environmental noise exposure changed the composition of the gut microbiome in both APP/PS1 and WT mice, which encoded functional categories including galactose and phospholipid metabolism, oxidative stress, and cell senescence
Sun *et al.* [101]	Male C57BL/6 mice divided into control group, Parkinson's disease (PD) group induced by 1-methyl-4-phenyl-1,2,3,6-tetrahydropyridine, and diseased group treated with *Clostridium butyricum* (*Cb*)	PD	*Cb* reversed the diseased mice’s dysbiosis of gut microbiota and exerted neuroprotective functions through the microbiota-gut-brain axis
Hill-Burns *et al.* [102]	PD cases and controls	PD	The abundance of the families *Bifidobacteriaceae*, *Christensenellaceae*, *Tissierellaceae*, *Lachnospiraceae*, *Lactobacillaceae*, *Pasteurellaceae*, and *Verrucomicrobiaceae* were significantly altered
Brenner *et al.* [103]	Amyotrophic lateral sclerosis (ALS) patients and healthy persons	ALS	ALS patients did not exhibit a substantial alteration of the gut microbiota composition
Kong *et al.* [104]	Male Huntington's disease (HD) mice and WT littermate controls	HD	There was an increase in *Bacteriodetes* and a proportional decrease in *Firmicutes* in the HD gut microbiome, which were connected with HD patients’ impaired weight gain and alterations in the gut microenvironment

Data obtained by 16S rRNA sequencing that emphasizes the specific constitution of intestinal microbiota in patients with neurodegenerative diseases.

Recently, amplicons have been inferred as amplicon sequence variants (ASVs) with different de-noising algorithms, such as Deblur [50] and DADA2 [51]. These methods attempt to infer the biological sequences by obtaining single-nucleotide resolution from Illumina data. Compared to OTUs, ASVs can capture all biological variation without the restriction of a reference database, and ASVs can be replicated and compared across different datasets [49]. ASVs are considered to make marker-gene sequencing more precise and reproducible. Thus, more sequencing methods are being developed.

### Whole-Genome Shotgun Sequencing (WGS)

WGS uses random primers to sequence overlapping regions of the genome. WGS can define taxa more accurately at the species level and has more advantages than 16S rRNA sequencing [52]. WGS enhances the detection range of bacterial diversity, improves the detection accuracy of species, estimates the functional potential of the microbiome, and identifies novel strains or mutations in samples [47, 52].

WGS has also been used to investigate the relationship between gut microbes and neurodegenerative diseases (Tables 2, 3, and Supplementary Table). WGS still has evident limitations that may make it unsuitable for large-scale studies. Obtaining sufficient sequencing depth can be costly, given that complex bacterial communities need high coverage and contain large amounts of non-target DNA, such as human DNA in human fecal samples. Besides, it is computationally challenging to remove redundant communities [53].

**Table 2:** Representative research using metagenomics sequencing to test stool samples

Reference	Subjects	Condition	Main findings
McCann *et al.* [105]	Elderly individuals with different levels of cognitive ability	Alzheimer's disease (AD)	Some intestinal microbes possessed the genes to produce vitamin K in the form of menaquinone (MK). Certain MK isoforms synthesized by the gut microbiome, particularly the longer chains, were positively associated with cognition
Qian *et al.* [106]	Idiopathic Chinese Parkinson’s disease (PD) patients and their healthy spouses living in the same household	PD	Potential diagnostic biomarkers of PD based on metagenomics sequencing results
Nicholson *et al.* [107]	Amyotrophic lateral sclerosis (ALS) patients and healthy controls (HC)	ALS	The relative abundance of the butyrate-producing bacteria *Eubacterium rectale* and *Roseburia intestinalis*, which play important roles in regulating inflammation and gut integrity, was lower in ALS patients than in HC
Wu *et al.* [108]	Healthy young, healthy elderly, and centenarians	Aging	The gut microbiota in centenarians was characterized by depletion of *Faecalibacterium prausnitzii* and *Eubacterium rectale* and enrichment of *Methanobrevibacter smithii* and *Bifidobacterium adolescentis*, compared with other groups. Functional analysis revealed that the microbiota in centenarians had a high capacity for glycolysis and fermentation to short-chain fatty-acids

Compared to 16S rRNA sequencing, researchers usually use metagenomics data for functional analysis, using databases such as Kyoto Encyclopedia of Genes and Genomes, in order to fully understand the biological meanings encoded in the genome.

**Table 3:** Representative research using multi-omics to test stool samples

Reference	Subjects	Approaches	Condition	Main findings
Peng *et al.* [109]	Senescence-accelerated mouse prone 8 (SAMP8) mice and senescence-accelerated mouse resistant 1 (SAMR1) mice	16S rRNA sequencing	Alzheimer's disease (AD)	The SAMP8 mice displayed a characteristic composition of the gut microbiome that clearly differed from that of the SAMR1 mice. Specifically, the relative abundance of the dominant genus in AD mice, *norank_f__Bacteroidales_S24-7_group*, was significantly decreased and the abundance of *unclassified_f__Lachnospiraceae* and *norank_f__Lachnospiraceae* were increased
		Metagenomics sequencing		Both Clusters of Orthologous Genes and Kyoto Encyclopedia of Genes and Genomes analyses indicated that alterations of gut microbiota contribute to AD pathogenesis through metabolic pathways
Heintz-Buschart *et al.* [110]	Parkinson's disease (PD) patients, idiopathic rapid eye movement (REM) sleep behavior disorder patients, and healthy controls	16S rRNA sequencing	PD	There was an 80% difference in gut microbes between PD and controls. Gut microbes in PD showed trends similar to idiopathic REM sleep behavior disorder patients, and were associated with non-motor symptoms
		Metagenomics sequencing		Metagenomics sequencing reconstructed genomes of so far uncharacterized differentially-abundant organisms
Tan *et al.* [111]	PD patients and healthy controls	16S rRNA sequencing	PD	The fecal microbiome in PD was significantly different from controls
		Metabolite analysis		The fecal metabolome composition in PD was significantly different from controls, whose predicted functions contained bioactive molecules with putative neuroprotective effects and other compounds involved in neurodegeneration
Hor *et al.* [112]	Male Sprague-Dawley rats divided into 6 groups: young rats as naïve control and D-galactose senescence-induced rats: (1) no intervention, (2) receiving *Lactobacillus fermentum DR9*, (3) receiving *L. paracasei OFS 0291*, (4) receiving *L. helveticus OFS 1515* and (5) receiving metformin	16S rRNA sequencing	Aging	After senescence induction, the ratio of *Firmicutes*/*Bacteroidetes* was significantly lowered, while *L. helveticus OFS 1515* and *L. fermentum DR9* increased the ratio. *L. paracasei OFS 0291* and *L. helveticus OFS 1515* reduced the opportunistic Bacteroides pathogens, while *L. fermentum DR9* promoted the proliferation of *Lactobacillus*.
		Metabolite analysis		D-gal-induced senescence had a great impact on amino-acid metabolism such as urocanic acid, citrulline, cystamine, and 5-oxoproline. *L. fermentum DR9* promoted antioxidative effects through upregulation of oxoproline, and both *L. paracasei OFS 0291* and *L. helveticus OFS 1515* restored the levels of reducing sugars, arabinose, and ribose similar to young rats

Multi-omics combines the advantages of different sequencing techniques and examines the data on gut microbiota from different angles.

### Metatranscriptomics

Metagenomics is incapable of elucidating functional interactions within complex microbial ecosystems or of assessing how these interactions vary with changing environments, including diet [54]. Metatranscriptomics based on next-generation sequencing can fill this gap and be used to analyze gene expression and evaluate microbial function directly from microbial combinations [55]. This sequencing method extracts all actively transcribed genes, not all existing genes, because some genes may not be sufficiently active at the time of sampling [56], and describes gene expression in microbial communities. This provides stronger evidence for the functional activity of genes than DNA-based community sequencing methods because many genes are conditionally expressed [57].

Currently, few studies of neurodegenerative diseases using metatranscriptomics are being reported. Chung *et al.* [58] examined the cecum contents of eight wild mice using metatranscriptomics and noted that genes for cofactors, amino-acid metabolism, and vitamin metabolism were upregulated in *Deferribacteraceae*, while genes for carbohydrate metabolism were upregulated in *Muribaculaceae*. Considering the rapid changes in the mRNA transcript pool, it remains uncertain whether the RNA recovered from feces can accurately represent intestinal activity processes, and not an outcome of sampling-induced stressful conditions [59].

### Metaproteomics

The presence of RNA measured by metatranscriptomics does not necessarily mean that the genes are expressed or translated into protein [60]. Since the purpose of metaproteomics analysis is to characterize the full protein content of environmental samples at a specific time [61], it is both cost-saving and provides direct insight into the functional information in environmental samples compared to metatranscriptomics [62]. Furthermore, because the metaproteome is more stable than the metatranscriptome, changes caused by sampling, such as low sampling temperature to prevent RNA translation, are less likely to occur [59].

There are currently few cases where metaproteomics has been used to study neurodegenerative diseases. Chen *et al.* [63] detected significant changes in the fecal microbiome in patients with major depression using comparative metaproteomics.

Problems that metaproteomics cannot address include the absence of a unified protocol for sample preparation, the inability to measure proteins in low abundance in complex microbial communities, and the shortage of effective bioinformatics tools [64].

### Metabolomics

Metabolomics enables an integrated systematic quantitative and qualitative analysis of all small molecule metabolites in a biological system [65, 66]. These metabolites can originate from microbial symbionts, hosts, environmental intake, or a combination of these sources [67]. Metabolites participate in processes such as gluconeogenesis, glycolysis, lipid metabolism, amino-acid metabolism, and the urea cycle. They have many biological functions and can be indicative reporters of physiology [68].

Metabolomics is being used to study the relationship between gut microbes and neurodegenerative diseases (Table 3 and Supplementary Table). Still, the use of metabolomics faces many challenges [68]. First, it is still difficult to find metabolic biomarkers due to the large number, the wide range in concentration, and wide chemical diversity of metabolites. Second, more work is needed to develop a reliable method for distillation of useful information from the massive amounts of metabolomics data. Third, improving the stability of sampling and specificity of disease diagnosis deserves deeper study.

### Multi-omics

In addition to using individual multi-omics technologies for research, now many studies increasingly integrate various multi-omics methods to better understand the function of gut microbiome. This approach allows researchers to integrate the advantages of various multi-omics sequencing technologies in order to understand the role of the intestinal microbiota in various physiological and pathological states in an in-depth and comprehensive manner, to identify potential biomarkers of diseases, and to pursue possible therapeutic approaches.

There are many reports using multi-omics technologies to explore the relationships between gut microbes and neurodegenerative diseases (Table 3 and Table S1). Of course, there are many limitations to the integration of multi-omics sequencing technologies [69, 70]. First of all, in view of the limitation of under-sampling in all multi-omics datasets, it is still a major challenge to interpret the data at multiple levels. Second, it is not a trivial task to integrate multi-omics datasets considering the increased diversity and complexity of the collected data. There is an urgent need for effective bioinformatics tools and advanced statistical methods. Third, it is currently still too expensive to carry out wide multiple multi-omics studies.

## Effects of Intestinal Microbiome-Based Therapies on Neurodegenerative Diseases

Microbiome-based therapies include probiotics, prebiotics, and synbiotics. Besides, antibiotics and fecal microbiota transplantation (FMT) can also be included in these therapies. Postbiotics are defined as functional microbial fermentation components, which are combined with nutritional components to promote health [71]. Postbiotics include metabolites, short-chain fatty-acids (SCFAs), functional proteins, teichoic acid, and peptidoglycan-derived muropeptides [71]. The role of microbiome-based therapies in regulating intestinal microbiota and preventing or suppressing the development of neurodegenerative diseases is becoming increasingly plausible in light of the growing evidence linking the intestinal microbiome to neurotoxins, inflammatory responses, and immune responses. However, few relevant clinical trials have been completed, and most of these have the disadvantage of a small sample size and short trial time; thus, the efficacy of microbiome-based therapies in neurodegenerative diseases remains be further validated.

### Alzheimer's Disease

Some studies have indicated that high intake of probiotics, prebiotics, and other nutrients decreases the risk of onset of AD [72]. It has been shown that probiotics and prebiotics promote the growth of *Bifidobacterium* and inhibit the growth of *Enterobacteriaceae* and thus improve adaptive immune responses and reduce inflammatory responses [73]. Some clinical trials have revealed the effectiveness of probiotics in regulating gut microbiota disorders, and in preventing or inhibiting cognitive or emotional disorders, suggesting the potential of probiotics for the treatment of AD [20, 74]. Akbari *et al.* [75] found that, compared to AD patients treated with normal milk, patients who consumed milk rich in multiple *Lactobacillus* and *Bifidobacterium* species showed a significant (*P* < 0.001) improvement in the mini-mental state examination (MMSE) score. Tamtaji *et al.* [76] noted that the MMSE score of a group receiving probiotics such as *Lactobacillus*, *Bifidobacterium*, and selenium was significantly increased (*P* < 0.001) compared to control groups receiving only selenium or placebo. Also, the group with probiotics plus selenium intake showed a significant (*P* < 0.001) reduction in serum high-sensitivity C-reactive protein (hs-CRP) and a significant (*P* = 0.001) increase in total antioxidant capacity and glutathione (GSH), compared to the group with selenium-only intake. Administration of ampicillin to rats leads to increased serum corticosterone and impaired spatial memory, which are common features of AD pathology [77]. On the contrary, administration of rifampicin to AD animal models leads to reduced brain levels of Aβ and inflammatory cytokines [78]. At present, antibiotics are not considered as therapeutic agents for AD. Objectively, Sun *et al.* [79] reported that FMT treatment improves the cognitive deficits of AD mice and reduces Aβ deposition in the brain. The efficacy of FMT in AD patients is still being explored. It is known that probiotics have anti-inflammatory effects [80], antioxidant effects [44, 81] and enhance cognitive function [75], but the specific mechanisms underlying its therapeutic properties still needs further study.

### Parkinson's Disease

Probiotics may ameliorate PD through a variety of mechanisms, including stabilizing symptoms of anxiety and depression, reducing symptoms of gastrointestinal complications, strengthening the integrity of intestinal epithelial cells, regulating immunity, and inhibiting the growth of pathogenic bacteria [24, 82–84]. Georgescu *et al.* [85] have shown that preparations containing *Lactobacillus acidophilus* and *Bifidobacterium infantis* can significantly (*P* < 0.0001) reduce abdominal pain and bloating in PD patients. Borzabadi *et al.* [86] found that treatment with 8×10^9^ CFU/day probiotics for 12 weeks downregulates the gene expression of tumor necrosis factor alpha (*P* = 0.04), interleukin-1 (IL-1) (*P* = 0.03), and IL-8 (*P < *0.001) in patients with PD. Tamtaji *et al.* [87] administered quadruple probiotic treatment or placebo for 12 weeks to PD patients and found decreases in the Movement Disorders Society-Unified PD Rating Scale, hs-CRP, and malondialdehyde, and increased GSH in the group consuming probiotics.

Research on prebiotics and synbiotics is more limited. Some studies have indicated that prebiotic fibers that generate butyrate may have beneficial effects in PD patients [24, 82, 88]. Barichella *et al.* [89] demonstrated that constipation in patients with PD can be improved by daily consumption of fermented milk containing prebiotic fiber and multiple probiotic strains. Further investigations are desirable to clarify the exact mechanisms and effects of microbiome-based therapies for PD since the relevant preclinical and clinical studies are still insufficient.

### Short-chain Fatty-acids and Neurodegenerative Diseases

**Fig. 2:**
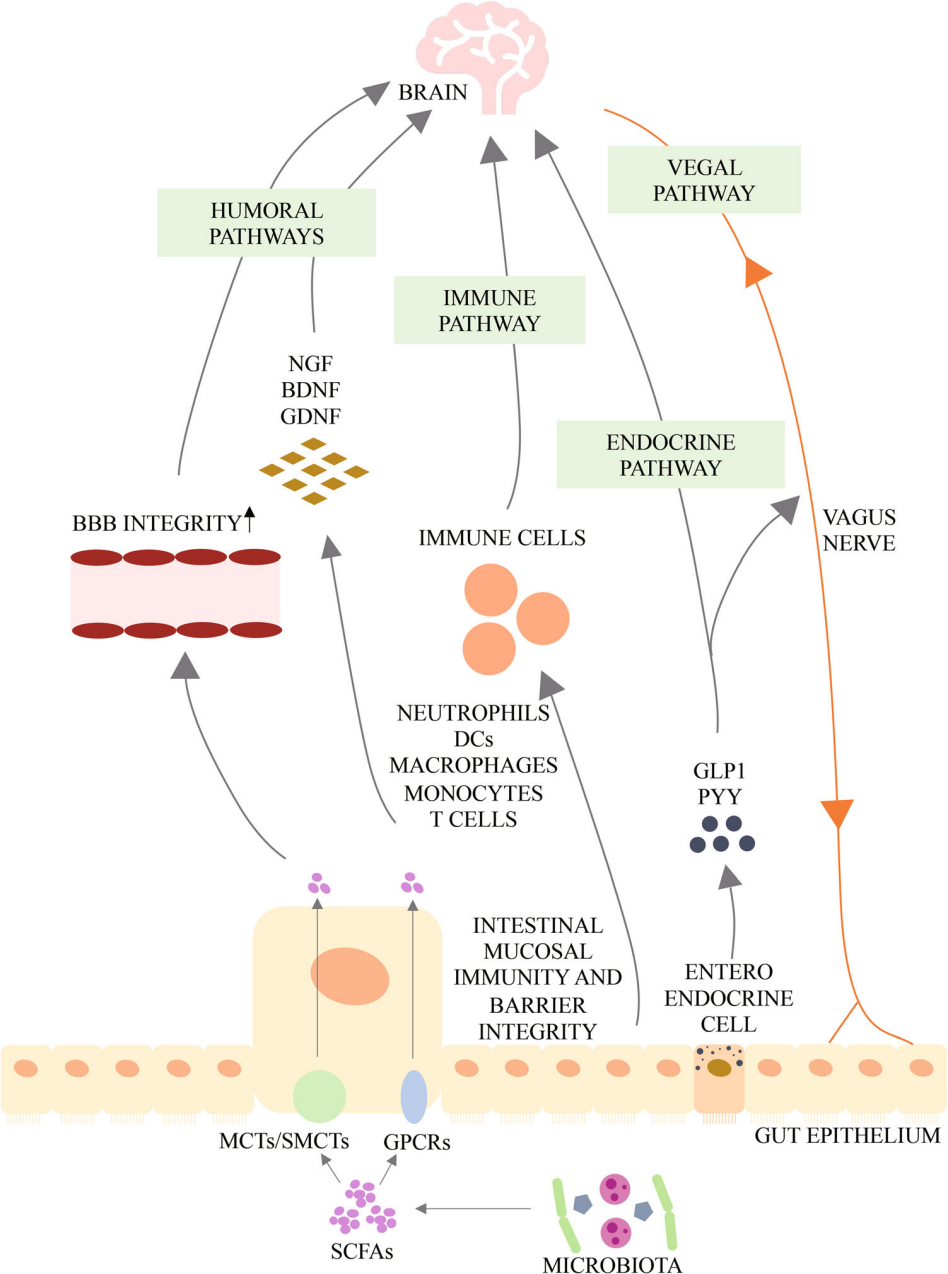
Potential pathways through which short chain fatty acids (SCFAs) modulate brain function. After production by gut microbiota, SCFAs are absorbed by colonocytes and any accessible cell through H^+^-dependent monocarboxylate transporters (MCTs) or sodium-dependent monocarboxylate transporters (SMCTs), or through binding to G protein coupled receptors (GPCRs) such as free fatty acid receptor 2 and 3 (FFAR2 and FFAR3), the GPCR109A and GPCR164. Intracellular SCFAs can inhibit the activity of histone deacetylases, preventing deacetylation of histones and leading to more transcriptionally active chromatin; or they can increase the activity of histone acetyltransferases, resulting in acetylation of histones and gene expression. SCFAs influence the communication between gut and brain, as well as brain function, either directly or indirectly via humoral, immune, endocrine and vagal pathways. Via the humoral route, SCFAs can cross the barrier or blood-brain barriers (BBB) via MCTs located on endothelial cells and influence BBB integrity by upregulating the expression of tight junction proteins. They can also modulate neurotrophic factors, such as nerve growth factor (NGF), brain-derived neurotrophic factor (BDNF), and glial cell line-derived neurotrophic factor (GDNF), and thus regulate the central nervous system and peripheral nervous system. Via the immune route, SCFAs can influence intestinal mucosal immunity by activating FFARs or by inhibiting deacetylation of histones. SCFAs can also enhance intestinal barrier integrity by upregulating the expression of tight junction proteins and augmenting transepithelial electrical resistance. In addition, SCFAs can regulate neutrophils, dendritic cells (DCs), macrophages and monocytes, and T cells, and thus maintain homeostasis. Via the endocrine route, SCFAs can interact with their receptors on enteroendocrine cells to induce the secretion of gut hormones such as glucagon-like peptide 1 (GLP1) and peptide YY (PYY), thus promoting indirect signaling to the brain via the systemic circulation or vagal pathways. SCFAs can also promote direct signaling to the brain via the vagal route. [90, 99]

The major metabolites arising from bacterial fermentation of dietary fiber in the intestines are SCFAs. Based on the available research results, SCFAs may affect the brain through direct humoral effects, indirect hormonal effects, immune pathways, and neural pathways, and may affect psychological function through interactions with G-protein-coupled receptors or histone deacetylases (Fig. 2) [90]. Evidence shows that SCFAs are associated with neurodegeneration [91]. Ho *et al.* found by *in vitro* experiments that selected SCFAs, especially butyric acid, valeric acid, and propionic acid, inhibit Aβ aggregation, which suggests the potential of SCFAs for treating AD. The role of SCFAs in PD is controversial [92]. Clinical studies have shown that PD patients have a lower abundance of SCFA-producing bacteria in the intestines [93] and lower fecal SCFA concentrations [94] than healthy controls. The plasma acetic acid concentration is significantly higher in PD patients than in controls [95]. Experiments have demonstrated that sodium butyrate rescues dopaminergic cells from α-syn-induced DNA damage [96]. In contrast, some experiments in animal models of PD show an increase of SCFAs in feces [97]. Oral administration of SCFA mixtures of acetate, butyrate, and propionate to PD mice that overexpress α-syn promote neuroinflammation and motor symptoms, suggesting that SCFAs aggravate symptoms associated with PD [5]. SCFAs could have either a beneficial or a harmful impact on neurodegenerative diseases, and the specific mechanisms underlying their effects on these diseases requires further study.

It has been demonstrated that microbiome-based therapies can be used to treat neurodegenerative diseases. It is of crucial importance to formulate an individualized treatment regimen to maximize their efficacy.

## Conclusions

In this review, we describe a large number of reports on the relationship between gut microbes and neurodegenerative diseases, focusing mainly on finding changes in the gut microbiota of people with these diseases, and on seeking improvement in disease progression with the use of probiotics. We also present some of the technical methods used to analyze the gut microbiota. However, the intrinsic link and detailed mechanisms between gut microbiota and neurodegenerative diseases, such as how gut microbiota cause disease development and how pathophysiological changes in the human body alter the stability of gut microbiota, is still being further explored and will remain a focus of future exploration. As technology advances, new techniques for studying the microbiome are constantly being developed and refined. In the near future, it is likely that the various multi-omics techniques will be further improved, costs will continually go down, and limitations will be gradually reduced. In this way, it is possible to combine a wide range of new technologies to assess the bi-directional links between neurodegenerative diseases and intestinal microbiota, and to discover their potential roles in the diagnosis, treatment, and prevention of these diseases, so as to effectively cure patients, reduce the physical and mental burdens on them and their families, slow down the aging process, and prolong human life.

## Supplementary Information

Below is the link to the electronic supplementary material.

Supplementary file1 (PDF 256 kb)

## References

[1] Dubinsky MC, Lin YC, Dutridge D, Picornell Y, Landers CJ, Farrior S (2006). Serum immune responses predict rapid disease progression among children with Crohn's disease: Immune responses predict disease progression. Am J Gastroenterol.

[2] Frank DN, St. Amand AL, Feldman RA, Boedeker EC, Harpaz N, Pace NR. Molecular-phylogenetic characterization of microbial community imbalances in human inflammatory bowel diseases. Proc Natl Acad Sci USA 2007, 104: 13780–13785. 10.1073/pnas.0706625104 PMC1959459 17699621

[3] O'Keefe SJ, Ou JH, Aufreiter S, O'Connor D, Sharma S, Sepulveda J (2009). Products of the colonic microbiota mediate the effects of diet on colon cancer risk. J Nutr.

[4] Hu X, Wang T, Jin F (2016). Alzheimer's disease and gut microbiota. Sci China Life Sci.

[5] Sampson TR, Debelius JW, Thron T, Janssen S, Shastri GG, Ilhan ZE (2016). Gut microbiota regulate motor deficits and neuroinflammation in a model of Parkinson's disease. Cell.

[6] Komaroff AL (2017). The microbiome and risk for obesity and diabetes. JAMA.

[7] Barlow GM, Yu A, Mathur R (2015). Role of the gut microbiome in obesity and diabetes mellitus. Nutr Clin Pract.

[8] Scher JU, Sczesnak A, Longman RS, Segata N, Ubeda C, Bielski C (2013). Expansion of intestinal *Prevotella copri* correlates with enhanced susceptibility to arthritis. Elife.

[9] Britton RA, Irwin R, Quach D, Schaefer L, Zhang J, Lee T (2014). Probiotic L. reuteri treatment prevents bone loss in a menopausal ovariectomized mouse model. J Cell Physiol.

[10] Vieira AT, Macia L, Galvão I, Martins FS, Canesso MC, Amaral FA (2015). A role for gut microbiota and the metabolite-sensing receptor GPR43 in a murine model of gout. Arthritis Rheumatol.

[11] Lynch SV, Pedersen O (2016). The human intestinal microbiome in health and disease. N Engl J Med.

[12] da Costa JP, Vitorino R, Silva GM, Vogel C, Duarte AC, Rocha-Santos T (2016). A synopsis on aging-Theories, mechanisms and future prospects. Ageing Res Rev.

[13] Marizzoni M, Provasi S, Cattaneo A, Frisoni GB (2017). Microbiota and neurodegenerative diseases. Curr Opin Neurol.

[14] Dinan TG, Cryan JF (2017). The microbiome-gut-brain axis in health and disease. Gastroenterol Clin North Am.

[15] O'Toole PW, Jeffery IB (2015). Gut microbiota and aging. Science.

[16] Kowalski K, Mulak A (2019). Brain-gut-microbiota axis in Alzheimer's disease. J Neurogastroenterol Motil.

[17] Jouanne M, Rault S, Voisin-Chiret AS (2017). Tau protein aggregation in Alzheimer's disease: An attractive target for the development of novel therapeutic agents. Eur J Med Chem.

[18] Köhler CA, Maes M, Slyepchenko A, Berk M, Solmi M, Lanctôt KL (2016). The gut-brain axis, including the microbiome, leaky gut and bacterial translocation: Mechanisms and pathophysiological role in Alzheimer's disease. Curr Pharm Des.

[19] Cattaneo A, Cattane N, Galluzzi S, Provasi S, Lopizzo N, Festari C (2017). Association of brain amyloidosis with pro-inflammatory gut bacterial taxa and peripheral inflammation markers in cognitively impaired elderly. Neurobiol Aging.

[20] Mancuso C, Santangelo R (2018). Alzheimer's disease and gut microbiota modifications: The long way between preclinical studies and clinical evidence. Pharmacol Res.

[21] Jiang C, Li G, Huang P, Liu Z, Zhao B (2017). The gut microbiota and Alzheimer's disease. J Alzheimers Dis.

[22] Haran JP, Bhattarai SK, Foley SE, Dutta P, Ward DV, Bucci V, *et al*. Alzheimer's disease microbiome is associated with dysregulation of the anti-inflammatory P-glycoprotein pathway. mBio 2019, 10: e00632–e00619. 10.1128/mBio.00632-19 PMC6509190 31064831

[23] Kim MS, Kim Y, Choi H, Kim W, Park S, Lee D (2020). Transfer of a healthy microbiota reduces amyloid and tau pathology in an Alzheimer's disease animal model. Gut.

[24] Caputi V, Giron MC (2018). Microbiome-gut-brain axis and toll-like receptors in Parkinson's disease. Int J Mol Sci.

[25] Perez-Pardo P, Kliest T, Dodiya HB, Broersen LM, Garssen J, Keshavarzian A (2017). The gut-brain axis in Parkinson's disease: Possibilities for food-based therapies. Eur J Pharmacol.

[26] Felice VD, Quigley EM, Sullivan AM, O'Keeffe GW, O'Mahony SM (2016). Microbiota-gut-brain signalling in Parkinson's disease: Implications for non-motor symptoms. Parkinsonism Relat Disord.

[27] Campos-Acuña J, Elgueta D, Pacheco R (2019). T-cell-driven inflammation as a mediator of the gut-brain axis involved in Parkinson's disease. Front Immunol.

[28] Scheperjans F, Aho V, Pereira PA, Koskinen K, Paulin L, Pekkonen E (2015). Gut microbiota are related to Parkinson's disease and clinical phenotype. Mov Disord.

[29] Mulak A, Bonaz B (2015). Brain-gut-microbiota axis in Parkinson's disease. World J Gastroenterol.

[30] Keshavarzian A, Green SJ, Engen PA, Voigt RM, Naqib A, Forsyth CB (2015). Colonic bacterial composition in Parkinson's disease. Mov Disord.

[31] Hou YF, Shan C, Zhuang SY, Zhuang QQ, Ghosh A, Zhu KC (2021). Gut microbiota-derived propionate mediates the neuroprotective effect of osteocalcin in a mouse model of Parkinson's disease. Microbiome.

[32] Roy Sarkar S, Banerjee S (2019). Gut microbiota in neurodegenerative disorders. J Neuroimmunol.

[33] Spielman LJ, Gibson DL, Klegeris A (2018). Unhealthy gut, unhealthy brain: The role of the intestinal microbiota in neurodegenerative diseases. Neurochem Int.

[34] Pellegrini C, Antonioli L, Colucci R, Blandizzi C, Fornai M (2018). Interplay among gut microbiota, intestinal mucosal barrier and enteric neuro-immune system: A common path to neurodegenerative diseases?. Acta Neuropathol.

[35] Fang X (2016). Potential role of gut microbiota and tissue barriers in Parkinson's disease and amyotrophic lateral sclerosis. Int J Neurosci.

[36] Blacher E, Bashiardes S, Shapiro H, Rothschild D, Mor U, Dori-Bachash M (2019). Potential roles of gut microbiome and metabolites in modulating ALS in mice. Nature.

[37] Bianchi VE, Herrera PF, Laura R. Effect of nutrition on neurodegenerative diseases. A systematic review. Nutr Neurosci 2019: 1–25. 10.1080/1028415X.2019.1681088 31684843

[38] Sharma S, Taliyan R (2015). Transcriptional dysregulation in Huntington's disease: The role of histone deacetylases. Pharmacol Res.

[39] Tremlett H, Bauer KC, Appel-Cresswell S, Finlay BB, Waubant E (2017). The gut microbiome in human neurological disease: A review. Ann Neurol.

[40] Kong G, Ellul S, Narayana VK, Kanojia K, Ha HTT, Li SS (2021). An integrated metagenomics and metabolomics approach implicates the microbiota-gut-brain axis in the pathogenesis of Huntington's disease. Neurobiol Dis.

[41] Franceschi C, Bonafè M, Valensin S, Olivieri F, de Luca M, Ottaviani E (2000). Inflamm-aging An evolutionary perspective on immunosenescence. Ann N Y Acad Sci.

[42] Frasca D, Diaz A, Romero M, Garcia D, Blomberg BB (2020). B Cell Immunosenescence. Annu Rev Cell Dev Biol.

[43] Borre YE, O'Keeffe GW, Clarke G, Stanton C, Dinan TG, Cryan JF (2014). Microbiota and neurodevelopmental windows: Implications for brain disorders. Trends Mol Med.

[44] Westfall S, Lomis N, Kahouli I, Dia SY, Singh SP, Prakash S (2017). Microbiome, probiotics and neurodegenerative diseases: Deciphering the gut brain axis. Cell Mol Life Sci.

[45] García-Peña C, Álvarez-Cisneros T, Quiroz-Baez R, Friedland RP (2017). Microbiota and Aging. Arch Med Res.

[46] Biagi E, Franceschi C, Rampelli S, Severgnini M, Ostan R, Turroni S (2016). Gut microbiota and extreme longevity. Curr Biol.

[47] Cryan JF, O'Riordan KJ, Cowan CSM, Sandhu KV, Bastiaanssen TFS, Boehme M (2019). The microbiota-gut-brain axis. Physiol Rev.

[48] Allali I, Arnold JW, Roach J, Cadenas MB, Butz N, Hassan HM (2017). A comparison of sequencing platforms and bioinformatics pipelines for compositional analysis of the gut microbiome. BMC Microbiol.

[49] Callahan BJ, McMurdie PJ, Holmes SP (2017). Exact sequence variants should replace operational taxonomic units in marker-gene data analysis. ISME J.

[50] Amir A, McDonald D, Navas-Molina JA, Kopylova E, Morton JT, Zech Xu Z, *et al*. Deblur rapidly resolves single-nucleotide community sequence patterns. mSystems 2017, 2: e00191–e00116. 10.1128/mSystems.00191-16 PMC5340863 28289731

[51] Callahan BJ, McMurdie PJ, Rosen MJ, Han AW, Johnson AJ, Holmes SP (2016). DADA2: High-resolution sample inference from Illumina amplicon data. Nat Methods.

[52] Ranjan R, Rani A, Metwally A, McGee HS, Perkins DL (2016). Analysis of the microbiome: Advantages of whole genome shotgun versus 16S amplicon sequencing. Biochem Biophys Res Commun.

[53] Mukherjee C, Beall CJ, Griffen AL, Leys EJ (2018). High-resolution ISR amplicon sequencing reveals personalized oral microbiome. Microbiome.

[54] Davids M, Hugenholtz F, Martins-dos-Santos V, Smidt H, Kleerebezem M, Schaap PJ (2016). Functional profiling of unfamiliar microbial communities using a validated de novo assembly metatranscriptome pipeline. PLoS One.

[55] Wu JY, Gao WM, Zhang WW, Meldrum DR (2011). Optimization of whole-transcriptome amplification from low cell density deep-sea microbial samples for metatranscriptomic analysis. J Microbiol Methods.

[56] Deng ZL, Gottschick C, Bhuju S, Masur C, Abels C, Wagner-Döbler I. Metatranscriptome analysis of the vaginal microbiota reveals potential mechanisms for protection against metronidazole in bacterial vaginosis. mSphere 2018, 3: e00262–e00218. 10.1128/mSphereDirect.00262-18 PMC5990888 29875146

[57] Berman HL, McLaren MR, Callahan BJ (2020). Understanding and interpreting community sequencing measurements of the vaginal microbiome. BJOG.

[58] Chung YW, Gwak HJ, Moon S, Rho M, Ryu JH (2020). Functional dynamics of bacterial species in the mouse gut microbiome revealed by metagenomic and metatranscriptomic analyses. PLoS One.

[59] Malan-Muller S, Valles-Colomer M, Raes J, Lowry CA, Seedat S, Hemmings SMJ (2018). The gut microbiome and mental health: Implications for anxiety- and trauma-related disorders. OMICS.

[60] Zhang X, Figeys D (2019). Perspective and guidelines for metaproteomics in microbiome studies. J Proteome Res.

[61] Snelling TJ, Wallace RJ (2017). The rumen microbial metaproteome as revealed by SDS-PAGE. BMC Microbiol.

[62] Lü F, Bize A, Guillot A, Monnet V, Madigou C, Chapleur O (2014). Metaproteomics of cellulose methanisation under thermophilic conditions reveals a surprisingly high proteolytic activity. ISME J.

[63] Chen Z, Li J, Gui SW, Zhou CJ, Chen JJ, Yang CC (2018). Comparative metaproteomics analysis shows altered fecal microbiota signatures in patients with major depressive disorder. Neuroreport.

[64] Zhang X, Li LY, Mayne J, Ning ZB, Stintzi A, Figeys D (2018). Assessing the impact of protein extraction methods for human gut metaproteomics. J Proteomics.

[65] Luan HM, Wang X, Cai ZW (2019). Mass spectrometry-based metabolomics: Targeting the crosstalk between gut microbiota and brain in neurodegenerative disorders. Mass Spectrom Rev.

[66] Smirnov KS, Maier TV, Walker A, Heinzmann SS, Forcisi S, Martinez I (2016). Challenges of metabolomics in human gut microbiota research. Int J Med Microbiol.

[67] Loftfield E, Vogtmann E, Sampson JN, Moore SC, Nelson H, Knight R (2016). Comparison of collection methods for fecal samples for discovery metabolomics in epidemiologic studies. Cancer Epidemiol Biomarkers Prev.

[68] Hyötyläinen T (2012). Novel methodologies in metabolic profiling with a focus on molecular diagnostic applications. Expert Rev Mol Diagn.

[69] Valles-Colomer M, Darzi Y, Vieira-Silva S, Falony G, Raes J, Joossens M (2016). Meta-omics in inflammatory bowel disease research: Applications, challenges, and guidelines. J Crohns Colitis.

[70] Zhang X, Li LY, Butcher J, Stintzi A, Figeys D (2019). Advancing functional and translational microbiome research using meta-omics approaches. Microbiome.

[71] Wegh CAM, Geerlings SY, Knol J, Roeselers G, Belzer C (2019). Postbiotics and their potential applications in early life nutrition and beyond. Int J Mol Sci.

[72] Pistollato F, Iglesias RC, Ruiz R, Aparicio S, Crespo J, Lopez LD (2018). Nutritional patterns associated with the maintenance of neurocognitive functions and the risk of dementia and Alzheimer's disease: A focus on human studies. Pharmacol Res.

[73] Pistollato F, Sumalla Cano S, Elio I, Masias Vergara M, Giampieri F, Battino M (2016). Role of gut microbiota and nutrients in amyloid formation and pathogenesis of Alzheimer disease. Nutr Rev.

[74] Angelucci F, Cechova K, Amlerova J, Hort J (2019). Antibiotics, gut microbiota, and Alzheimer's disease. J Neuroinflammation.

[75] Akbari E, Asemi Z, Daneshvar Kakhaki R, Bahmani F, Kouchaki E, Tamtaji OR (2016). Effect of probiotic supplementation on cognitive function and metabolic status in Alzheimer's disease: A randomized, double-blind and controlled trial. Front Aging Neurosci.

[76] Tamtaji OR, Heidari-Soureshjani R, Mirhosseini N, Kouchaki E, Bahmani F, Aghadavod E (2019). Probiotic and selenium co-supplementation, and the effects on clinical, metabolic and genetic status in Alzheimer's disease: A randomized, double-blind, controlled trial. Clin Nutr.

[77] Wang T, Hu X, Liang S, Li W, Wu X, Wang L (2015). *Lactobacillus fermentum* NS_9_ restores the antibiotic induced physiological and psychological abnormalities in rats. Benef Microbes.

[78] Yulug B, Hanoglu L, Ozansoy M, Isık D, Kilic U, Kilic E (2018). Therapeutic role of rifampicin in Alzheimer's disease. Psychiatry Clin Neurosci.

[79] Sun J, Xu J, Ling Y, Wang F, Gong T, Yang C (2019). Fecal microbiota transplantation alleviated Alzheimer’s disease-like pathogenesis in APP/PS1 transgenic mice. Transl Psychiatry.

[80] Leblhuber F, Steiner K, Schuetz B, Fuchs D, Gostner JM (2018). Probiotic supplementation in patients with Alzheimer's dementia - an explorative intervention study. Curr Alzheimer Res.

[81] Bonfili L, Cecarini V, Cuccioloni M, Angeletti M, Berardi S, Scarpona S (2018). SLAB51 probiotic formulation activates SIRT1 pathway promoting antioxidant and neuroprotective effects in an AD mouse model. Mol Neurobiol.

[82] Parisa Gazerani (2019). Probiotics for Parkinson’s Disease. Int J Mol Sci.

[83] Fang X (2019). Microbial treatment: The potential application for Parkinson's disease. Neurol Sci.

[84] Dutta SK, Verma S, Jain V, Surapaneni BK, Vinayek R, Phillips L (2019). Parkinson's disease: The emerging role of gut dysbiosis, antibiotics, probiotics, and fecal microbiota transplantation. J Neurogastroenterol Motil.

[85] Georgescu D, Ancusa OE, Georgescu LA, Ionita I, Reisz D (2016). Nonmotor gastrointestinal disorders in older patients with Parkinson's disease: Is there hope?. Clin Interv Aging.

[86] Borzabadi S, Oryan S, Eidi A, Aghadavod E, Daneshvar Kakhaki R, Tamtaji OR (2018). The effects of probiotic supplementation on gene expression related to inflammation, insulin and lipid in patients with Parkinson's disease: A randomized, double-blind. PlaceboControlled trial. Arch Iran Med.

[87] Tamtaji OR, Taghizadeh M, Daneshvar Kakhaki R, Kouchaki E, Bahmani F, Borzabadi S (2019). Clinical and metabolic response to probiotic administration in people with Parkinson's disease: A randomized, double-blind, placebo-controlled trial. Clin Nutr.

[88] Cantu-Jungles TM, Rasmussen HE, Hamaker BR (2019). Potential of prebiotic butyrogenic fibers in Parkinson's disease. Front Neurol.

[89] Barichella M, Pacchetti C, Bolliri C, Cassani E, Iorio L, Pusani C (2016). Probiotics and prebiotic fiber for constipation associated with Parkinson disease: An RCT. Neurology.

[90] Dalile B, Van Oudenhove L, Vervliet B, Verbeke K (2019). The role of short-chain fatty acids in microbiota–gut–brain communication. Nat Rev Gastroenterol Hepatol.

[91] Bruning J, Chapp A, Kaurala GA, Wang RJ, Techtmann S, Chen QH (2020). Gut microbiota and short chain fatty acids: Influence on the autonomic nervous system. Neurosci Bull.

[92] Ho L, Ono K, Tsuji M, Mazzola P, Singh R, Pasinetti GM (2018). Protective roles of intestinal microbiota derived short chain fatty acids in Alzheimer's disease-type beta-amyloid neuropathological mechanisms. Expert Rev Neurother.

[93] Li W, Wu XL, Hu X, Wang T, Liang S, Duan YF (2017). Structural changes of gut microbiota in Parkinson's disease and its correlation with clinical features. Sci China Life Sci.

[94] Unger MM, Spiegel J, Dillmann KU, Grundmann D, Philippeit H, Bürmann J (2016). Short chain fatty acids and gut microbiota differ between patients with Parkinson's disease and age-matched controls. Parkinsonism Relat Disord.

[95] Shin C, Lim Y, Lim H, Ahn TB (2020). Plasma short-chain fatty acids in patients with Parkinson's disease. Mov Disord.

[96] Paiva I, Pinho R, Pavlou MA, Hennion M, Wales P, Schütz AL (2017). Sodium butyrate rescues dopaminergic cells from alpha-synuclein-induced transcriptional deregulation and DNA damage. Hum Mol Genet.

[97] Sun MF, Zhu YL, Zhou ZL, Jia XB, Xu YD, Yang Q (2018). Neuroprotective effects of fecal microbiota transplantation on MPTP-induced Parkinson's disease mice: Gut microbiota, glial reaction and TLR4/TNF-α signaling pathway. Brain Behav Immun.

[98] Burokas A, Moloney RD, Dinan TG, Cryan JF (2015). Microbiota regulation of the Mammalian gut-brain axis. Adv Appl Microbiol.

[99] Silva YP, Bernardi A, Frozza RL (2020). The role of short-chain fatty acids from gut microbiota in gut-brain communication. Front Endocrinol (Lausanne).

[100] Chi HM, Cao W, Zhang M, Su DH, Yang HL, Li Z (2021). Environmental noise stress disturbs commensal microbiota homeostasis and induces oxi-inflammmation and AD-like neuropathology through epithelial barrier disruption in the EOAD mouse model. J Neuroinflammation.

[101] Sun J, Li HJ, Jin YJ, Yu JH, Mao SY, Su KP (2021). Probiotic *Clostridium butyricum* ameliorated motor deficits in a mouse model of Parkinson's disease via gut microbiota-GLP-1 pathway. Brain Behav Immun.

[102] Hill-Burns EM, Debelius JW, Morton JT, Wissemann WT, Lewis MR, Wallen ZD (2017). Parkinson's disease and Parkinson's disease medications have distinct signatures of the gut microbiome. Mov Disord.

[103] Brenner D, Hiergeist A, Adis C, Mayer B, Gessner A, Ludolph AC (2018). The fecal microbiome of ALS patients. Neurobiol Aging.

[104] Kong G, Cao KL, Judd LM, Li SS, Renoir T, Hannan AJ. Microbiome profiling reveals gut dysbiosis in a transgenic mouse model of Huntington's disease. Neurobiol Dis 2020, 135: 104268. 10.1016/j.nbd.2018.09.001 30194046

[105] McCann A, Jeffery IB, Ouliass B, Ferland G, Fu X, Booth SL (2019). Exploratory analysis of covariation of microbiota-derived vitamin K and cognition in older adults. Am J Clin Nutr.

[106] Qian YW, Yang XD, Xu SQ, Huang P, Li BY, Du JJ (2020). Gut metagenomics-derived genes as potential biomarkers of Parkinson's disease. Brain.

[107] Nicholson K, Bjornevik K, Abu-Ali G, Chan J, Cortese M, Dedi B (2021). The human gut microbiota in people with amyotrophic lateral sclerosis. Amyotroph Lateral Scler Frontotemporal Degener.

[108] Wu L, Zeng TS, Zinellu A, Rubino S, Kelvin DJ, Carru C (2019). A cross-sectional study of compositional and functional profiles of gut microbiota in Sardinian centenarians. mSystems.

[109] Peng WJ, Yi PJ, Yang JJ, Xu PP, Wang Y, Zhang ZY (2018). Association of gut microbiota composition and function with a senescence-accelerated mouse model of Alzheimer's Disease using 16S rRNA gene and metagenomic sequencing analysis. Aging (Albany NY).

[110] Heintz-Buschart A, Pandey U, Wicke T, Sixel-Döring F, Janzen A, Sittig-Wiegand E (2018). The nasal and gut microbiome in Parkinson's disease and idiopathic rapid eye movement sleep behavior disorder. Mov Disord.

[111] Tan AH, Chong CW, Lim SY, Yap IKS, Teh CSJ, Loke MF (2021). Gut microbial ecosystem in parkinson disease: New clinicobiological insights from multi-omics. Ann Neurol.

[112] Hor YY, Lew LC, Jaafar MH, Lau AS, Ong JS, Kato T (2019). Lactobacillus sp improved microbiota and metabolite profiles of aging rats. Pharmacol Res.

