# Supplementary material for: The Relationship Between the Gut Microbiome and Neurodegenerative Diseases
Source: Neurosci Bull. 2021 Jul 3;37(10):1510–22. doi: 10.1007/s12264-021-00730-8 (PMC8490573; doi:10.1007/s12264-021-00730-8)
Supplement: Supplementary file 1 — Supplementary file1 (PDF 256 kb) [file 12264_2021_730_MOESM1_ESM.pdf]

Supplementary Materials

Table S1 Research using different sequencing approaches.

Reference	Subjects	Approaches	Condition	Main findings
Shen *et al.* [1]	Wild-type (WT) and APPswe/PS1dE9 (APP/PS1) transgenic (Tg) Alzheimer's disease (AD) mice	16S amplicon sequencing	AD	The abundance of *Helicobacteraceae* and *Desulfovibrionaceae* at the family level and *Odoribacter* and *Helicobacter* at the genus level were significantly higher, while *Prevotella* abundance was significantly lower in APP/PS1 mice than in WT mice.
Zhuang *et al.* [2]	AD patients and healthy controls	16S amplicon sequencing	AD	The composition of the gut microbiota *Bacteroides*, *Actinobacteria*, *Ruminococcus*, *Lachnospiraceae*, and *Selenomonadales* differed between AD patients and controls.
Minter *et al.* [3]	Different drug delivery time of combinatorial antibiotic (ABX)	16S amplicon sequencing	AD	Early postnatal ABX treatment resulted in long-term alteration of gut microbial genera, predominantly expansion

	treatment in APP/PS1 mice			of *Lachnospiraceae* and reduction of S24-7.
Sun *et al.* [4]	Male APP/PS1 transgenic mice and matched WT mice treated with or without prebiotic fructooligosaccharides (FOS)	16S amplicon sequencing	AD	FOS reversed the altered microbial composition in the Tg group: increased *Proteobacteria*, *Helicobacteraceae* and *Desulfovibrionaceae*, and decreased *Lactobacillus*.
Parikh *et al.* [5]	Mice homozygous for apolipoprotein E 2 (APOE2), APOE3, or APOE4, and familial AD mutations	16S amplicon sequencing	AD	APOE alleles constitute a major genetic risk factor for AD. The relative abundance of multiple bacterial taxa was significantly different as a function of APOE genotype.
Scheperjans *et al.* [6]	Parkinson's disease (PD) patients and control subjects	16S amplicon sequencing	PD	The abundance of *Prevotellaceae* in feces of PD patients was reduced compared to controls.
Aho *et al.* [7]	PD patients and control subjects	16S amplicon sequencing	PD	Specific bacterial taxa differed between patients and controls, such as *Roseburia*, *Prevotella*, and *Bifidobacterium*.
Li *et al.* [8]	PD patients and healthy volunteers	16S amplicon sequencing	PD	Compared to healthy controls, putative cellulose- degrading bacteria from the genera *Blautia*, *Faecalibacterium*, and

				Ruminococcus were significantly decreased and putative pathobionts from the genera *Escherichia*/*Shigella*, *Streptococcus*, *Proteus*, and *Enterococcus* were significantly increased in PD patients.
Qian *et al.* [9]	PD patients and their healthy spouses	16S amplicon sequencing	PD	*Clostridium IV*, *Aquabacterium*, *Holdemania*, *Sphingomonas*, *Clostridium XVIII*, *Butyricicoccus*, and *Anaerotruncus* were enriched in the feces of PD patients.
Lin *et al.* [10]	PD patients and age-matched controls	16S amplicon sequencing	PD	The abundance of *Lachnospiraceae* was reduced and *Bifidobacteriaceae* was enriched in PD patients.
Lin *et al.* [11]	PD patients and age- and gender-matched controls	16S amplicon sequencing	PD	Increased relative abundance of *Verrucomicrobia*, *Mucispirillum*, *Porphyromonas*, *Lactobacillus*, and *Parabacteroides* in PD *vs* controls.
Mertsalmi *et al.* [12]	PD patients and controls	16S amplicon sequencing	PD	PD patients with IBS-like symptoms had lower abundance of *Prevotella* bacteria.

Keshavarzian *et al.* [13]	PD patients and healthy controls	16S amplicon sequencing	PD	Compared with PD, butyrate-producing bacteria from the genera *Blautia*, *Coprococcus*, and *Roseburia* were significantly increased in feces of controls and bacteria from the genus *Faecalibacterium* were significantly increased in the mucosa of controls.
Pietrucci *et al.* [14]	PD patients and healthy controls	16S amplicon sequencing	PD	Compared to healthy controls, the presence of the Lactobacillaceae, Enterobacteriaceae, and Enterococcaceae families were significantly higher, while Lachnospiraceae were significantly reduced in feces from PD patients.
Radulescu *et al.* [15]	WT and bacterial artificial chromosome model of HD (BACHD) Tg mice	16S amplicon sequencing	HD	Compared to WT mice, both 3- and 6 -month-old BACHD mice showed decreased abundance of *Prevotella* and *Bacteroides* at the genera level.
Park *et al.* [16]	Individuals living in urbanized town communities (UTC) and longevity village communities (LVC)	16S amplicon sequencing	Aging	Anti-inflammatory bacteria *Faecalibacterium spp. EF402172_s*, and *EF404388_s* were only detected in LVC, which may play an important role in preserving residents'

				health in LVC.
Smith *et al.* [17]	Male and female mice in control and acarbose treatment groups at each of three study sites	16S amplicon sequencing	Aging	This study suggested a role of the gut microbiota in the longevity-enhancing properties of acarbose.
Haran *et al.* [18]	Nursing home elders	Metagenomics sequencing	AD	The microbiome of AD elderly showed a lower proportion and prevalence of bacteria with the potential to synthesize butyrate, as well as higher abundances of taxa that are known to cause pro-inflammatory states.
Bedarf *et al.* [19]	Early stage, Levodopa-naïve PD patients and age-matched controls	Metagenomics sequencing	PD	*Akkermansia muciniphila* and unclassified *Firmicutes* were increased, while *Prevotella copri* and *Eubacterium biforme* were decreased in PD samples. Functional analyses of the metagenomes revealed metabolic differences in microbiota in PD involving ß-glucuronate and tryptophan metabolism.
Vulevic *et al.* [20]	Elderly volunteers assigned to two groups: one with placebo (maltodextrin)	Metabolite analysis	Aging	B-GOS led to increases in *Bifidobacteria*, which correlated with increased lactic acid in fecal fluid.

	and the other with Bimuno-galacto-oligosaccharides mixture (B-GOS)			
Gutiérrez-Díaz *et al.* [21]	Healthy mature volunteers from Asturias region (North Spain)	Metabolite analysis	Aging	Subjects on a Mediterranean diet presented greater fecal concentrations of benzoic and 3-hydroxyphenylacetic acids, and higher levels of *Clostridium cluster XVIa* and *Faecalibacterium prausnitzii*.
Conte *et al.* [22]	Caucasian volunteers divided into four groups: young subjects, elderly controls, elderly centenarians' offspring, and centenarians	Metabolite analysis	Aging	The existence of age-related differences in the pattern of fecal volatile organic compounds (VOCs) like formic acid and butyl ester contributes to explaining the complex biology of human aging and longevity. VOCs could be considered as promising markers to evaluate aging and longevity.
Brasili *et al.* [23]	Adult (3 months old) and aged (16 months old) mice received an oral supplementation of 2 probiotics or	Metabolite analysis	Aging	The probiotics induced in both adult and aged mice higher 4-hydroxyphenylacetate and lower xylose in treated mice compared with controls, while valerate was higher in treated

	phosphate-buffered saline daily for 30 days			adult mice and lower in treated aged mice than in controls.
Fujii *et al.* [24]	Mice transplanted with feces from AD patients or from healthy people	16S amplicon sequencing	AD	Microbial diversity was lower in healthy mice than in AD mice, but stable over time.
		Metabolite analysis		Nervous system-related metabolites, including taurine, γ-aminobutyrate, and valine, were significantly deficient in feces from mice transplanted with feces from AD patients compared with mice transplanted with feces from healthy people.
Ghaisas *et al.* [25]	MitoPark (MP) model mice with PD and their littermate controls (LCs)	16S amplicon sequencing	PD	A trend of higher *Prevotella* at ages 20 and 24 weeks in MP mice than in age-matched LC samples.
		Metabolite analysis		An increase in sterol absorption in 20-week-old MP mice compared to LCs supports the notion that this increased absorption may be a response to the rapid weight loss in MP mice after age 16 weeks, which corresponded to PD

				progression and severity.
Vemuri *et al.* [26]	Young and aging mice both fed with normal chow and probiotic chow containing *Lactobacillus acidophilus DDS-1*	16S amplicon sequencing	Aging	*DDS-1* increased *Akkermansia muciniphila* and *Lactobacillus spp.*, and reduced the abundance of *Proteobacteria spp.*.
		Metabolite analysis		*DDS-1* resulted in improvement of metabolic phenotype in the aging mice, involving amino-acid metabolism, protein synthesis and metabolism, carbohydrate metabolism, and butanoate metabolism.
Shenghua *et al.* [27]	22-month-old natural aging model divided into a model group and a FufangZhenzhuTiaoZhi (FTZ) group; two-month-old young mice as control	16S amplicon sequencing	Aging	FTZ treatment restored the intestinal microbiome disorders in aging mice, such as decreased beneficial butyrate-producing bacteria and increased harmful bacteria
		Metabolite analysis		FTZ treatment partially reversed fecal metabolite abnormalities, like linoleic acid, glycerophospholipid, α-linolenic acid, biosynthesis of unsaturated fatty acids, and

				glycerolipid metabolism.
Tuikhar *et al.* [28]	Centenarians (~100 years) and young adults (25–45 years) from a region with high centenarian prevalence (>6 centenarians/10,000 citizens) and young adults from a nearby region with low centenarian prevalence (<1 centenarian/10,000 citizens)	16S amplicon sequencing	Aging	There was a higher species richness and biodiversity in the family *Ruminococcaceae* in centenarians.
		Metabolite analysis		In centenarians, there was a higher level of compounds with neuro-pharmacological properties like GABA and imidazole 4-acetic acid, as well as azole compounds with antifungal and amebicidal activity, and lower levels of cyclohexanecarboxylic acid as an environmental contaminant in fecal extracts.

References

[1] Shen L, Liu L, Ji H-F. Alzheimer's Disease Histological and Behavioral Manifestations in Transgenic Mice Correlate with Specific Gut Microbiome State. Journal of Alzheimer's disease 2017, 56: 385-390.

[2] Zhuang Z-Q, Shen L-L, Li W-W, Fu X, Zeng F, Gui L*, et al.* Gut Microbiota is Altered in Patients with Alzheimer’s Disease. Journal of Alzheimer's Disease 2018, 63: 1337-1346.

[3] Minter MR, Hinterleitner R, Meisel M, Zhang C, Leone V, Zhang X*, et al.* Antibiotic-induced perturbations in microbial diversity during post-natal development alters amyloid pathology in an aged APPSWE/PS1ΔE9 murine model of Alzheimer's disease. Scientific Reports 2017, 7: 10411.

[4] Sun J, Liu S, Ling Z, Wang F, Ling Y, Gong T*, et al.* Fructooligosaccharides Ameliorating Cognitive Deficits and Neurodegeneration in APP/PS1 Transgenic Mice through Modulating Gut Microbiota. Journal of Agricultural and Food Chemistry 2019, 67: 3006-3017.

[5] Parikh IJ, Estus JL, Zajac DJ, Malik M, Maldonado Weng J, Tai LM*, et al.* Murine Gut Microbiome Association With APOE Alleles. Frontiers in Immunology 2020, 11: 200.

[6] Scheperjans F, Aho V, Pereira PAB, Koskinen K, Paulin L, Pekkonen E*, et al.* Gut microbiota are related to Parkinson's disease and clinical phenotype. Movement Disorders 2015, 30: 350-358.

[7] Aho VTE, Pereira PAB, Voutilainen S, Paulin L, Pekkonen E, Auvinen P*, et al.* Gut microbiota in Parkinson's disease: Temporal stability and relations to disease progression. EBioMedicine 2019, 44: 691-707.

[8] Li W, Wu X, Hu X, Wang T, Liang S, Duan Y*, et al.* Structural changes of gut microbiota in Parkinson’s disease and its correlation with clinical features. Science China Life Sciences

2017, 60: 1223-1233.

[9] Qian Y, Yang X, Xu S, Wu C, Song Y, Qin N*, et al.* Alteration of the fecal microbiota in Chinese patients with Parkinson's disease. Brain, Behavior, and Immunity 2018, 70: 194-202.

[10] Lin A, Zheng W, He Y, Tang W, Wei X, He R*, et al.* Gut microbiota in patients with Parkinson's disease in southern China. Parkinsonism & Related Disorders 2018, 53: 82-88.

[11] Lin C-H, Chen C-C, Chiang H-L, Liou J-M, Chang C-M, Lu T-P*, et al.* Altered gut microbiota and inflammatory cytokine responses in patients with Parkinson's disease. Journal of Neuroinflammation 2019, 16: 129.

[12] Mertsalmi TH, Aho VTE, Pereira PaB, Paulin L, Pekkonen E, Auvinen P*, et al.* More than constipation - bowel symptoms in Parkinson's disease and their connection to gut microbiota. European Journal of Neurology 2017, 24: 1375-1383.

[13] Keshavarzian A, Green SJ, Engen PA, Voigt RM, Naqib A, Forsyth CB*, et al.* Colonic bacterial composition in Parkinson's disease. Movement Disorders 2015, 30: 1351-1360.

[14] Pietrucci D, Cerroni R, Unida V, Farcomeni A, Pierantozzi M, Mercuri NB*, et al.* Dysbiosis of gut microbiota in a selected population of Parkinson's patients. Parkinsonism & Related Disorders 2019, 65: 124-130.

[15] Radulescu CI, Garcia-Miralles M, Sidik H, Bardile CF, Yusof NABM, Lee HU*, et al.* Manipulation of microbiota reveals altered callosal myelination and white matter plasticity in a model of Huntington disease. Neurobiology of Disease 2019, 127: 65-75.

[16] Park S-H, Kim K-A, Ahn Y-T, Jeong J-J, Huh C-S, Kim D-H. Comparative analysis of gut microbiota in elderly people of urbanized towns and longevity villages. BMC microbiology 2015, 15: 49.

[17] Smith BJ, Miller RA, Ericsson AC, Harrison DC, Strong R, Schmidt TM. Changes in the gut microbiome and fermentation products concurrent with enhanced longevity in acarbose-treated mice. BMC microbiology 2019, 19: 130.

[18] Haran JP, Bhattarai SK, Foley SE, Dutta P, Ward DV, Bucci V*, et al.* Alzheimer's Disease Microbiome Is Associated with Dysregulation of the Anti-Inflammatory P-Glycoprotein Pathway. mBio 2019, 10: e00632-00619.

[19] Bedarf JR, Hildebrand F, Coelho LP, Sunagawa S, Bahram M, Goeser F*, et al.* Functional implications of microbial and viral gut metagenome changes in early stage L-DOPA-naïve Parkinson's disease patients. Genome Medicine 2017, 9: 39.

[20] Vulevic J, Juric A, Walton GE, Claus SP, Tzortzis G, Toward RE*, et al.* Influence of galacto-oligosaccharide mixture (B-GOS) on gut microbiota, immune parameters and metabonomics in elderly persons. The British Journal of Nutrition 2015, 114: 586-595.

[21] Gutiérrez-Díaz I, Fernández-Navarro T, Salazar N, Bartolomé B, Moreno-Arribas MV, de Andres-Galiana EJ*, et al.* Adherence to a Mediterranean Diet Influences the Fecal Metabolic Profile of Microbial-Derived Phenolics in a Spanish Cohort of Middle-Age and Older People. Journal of Agricultural and Food Chemistry 2017, 65: 586-595.

[22] Conte M, Conte G, Martucci M, Monti D, Casarosa L, Serra A*, et al.* The smell of longevity: a combination of Volatile Organic Compounds (VOCs) can discriminate centenarians and their offspring from age-matched subjects and young controls. GeroScience 2020, 42: 201-216.

[23] Brasili E, Mengheri E, Tomassini A, Capuani G, Roselli M, Finamore A*, et al.* Lactobacillus acidophilus La5 and Bifidobacterium lactis Bb12 induce different age-related metabolic profiles revealed by 1H-NMR spectroscopy in urine and feces of mice. The Journal

of Nutrition 2013, 143: 1549-1557.

[24] Fujii Y, Nguyen TTT, Fujimura Y, Kameya N, Nakamura S, Arakawa K, *et al.* Fecal metabolite of a gnotobiotic mouse transplanted with gut microbiota from a patient with Alzheimer's disease. Bioscience, Biotechnology, and Biochemistry 2019, 83: 2144-2152.

[25] Ghaisas S, Langley MR, Palanisamy BN, Dutta S, Narayanaswamy K, Plummer PJ, *et al.* MitoPark transgenic mouse model recapitulates the gastrointestinal dysfunction and gut-microbiome changes of Parkinson's disease. NeuroToxicology 2019, 75: 186-199.

[26] Vemuri R, Shinde T, Gundamaraju R, Gondalia SV, Karpe AV, Beale DJ, *et al.* Lactobacillus acidophilus DDS-1 Modulates the Gut Microbiota and Improves Metabolic Profiles in Aging Mice. Nutrients 2018, 10: 1255.

[27] Shenghua P, Ziqin Z, Shuyu T, Huixia Z, Xianglu R, Jiao G. An integrated fecal microbiome and metabolome in the aged mice reveal anti-aging effects from the intestines and biochemical mechanism of FuFang zhenshu TiaoZhi(FTZ). Biomedicine & Pharmacotherapy 2020, 121: 109421.

[28] Tuikhar N, Keisam S, Labala RK, Imrat, Ramakrishnan P, Arunkumar MC, *et al.* Comparative analysis of the gut microbiota in centenarians and young adults shows a common signature across genotypically non-related populations. Mechanisms of Ageing and Development 2019, 179: 23-35.
